# Healthcare utilization and indirect cost of treatment associated with severe allergic asthma in a real-world setting

**DOI:** 10.1186/2045-7022-3-S1-P1

**Published:** 2013-05-03

**Authors:** Gert-Jan Braunstahl, Marco Deenstra, Janice Canvin, Guy Peachey, Chien-Wei Chen, Panayiotis Georgiou, Robert Maykut

**Affiliations:** 1 St Franciscus Gasthuis, Department of Pulmonary Medicine, India; 2 Flevoziekenhuis, Department of Pulmonology, the Netherlands; 3 Novartis Pharmaceuticals UK Limited, Clinical Development, UK; 4 Novartis Pharmaceuticals Corporation, Clinical Development, USA; 5 Novartis Pharma AG, Clinical Development, Switzerland

## Background

With an estimated 300 million individuals affected worldwide, asthma is associated with substantial social and economic burden. The cost of treating uncontrolled severe allergic asthma (SAA) is high encompassing a variety of direct medical costs and indirect costs. We present data on real-world healthcare utilization (direct) and school/work absence (indirect) in uncontrolled SAA patients receiving omalizumab in the eXpeRience registry.

## Methods

eXpeRience was a 2-year, global, single-arm, observational registry. Data were collected on real-world effectiveness, safety and use of omalizumab in patients with uncontrolled SAA. Asthma-related healthcare utilization (hospitalizations, emergency room visits or unscheduled doctor visits) and number of days missed from school/work were recorded.

## Results

The intent-to-treat population comprised 916 (97.1%) patients. Compared with the pre-treatment period, there were reductions in healthcare utilization and school/work absence after 12 and 24 months of omalizumab treatment.

**Table 1:** 

Variable, mean (SD); n	Pre-treatment**°** (N=916)	12 months (N=734)	24 months (N=643)
Asthma-related hospitalizations	0.7 (1.32); 882	0.1 (0.43); 702	0.1 (0.41); 628
Duration of hospitalization stay due to asthma, days	5.3 (11.05); 852	0.7 (3.84); 703	0.5 (3.39); 628
Asthma-related emergency room visits	1.8 (2.87); 867	0.2 (0.64); 700	0.1 (0.32); 627
Unscheduled asthma-related doctor visits	3.8 (4.79); 823	0.7 (1.43); 684	0.4 (0.99); 619
Asthma-related medical healthcare uses*	6.2 (6.97); 811	1.0 (1.96); 684	0.5 (1.28); 618
Absence from work due to asthma^#^, days	26.4 (49.61); 347	3.5 (17.28); 295	1.0 (4.66); 296
Absence from school due to asthma^#^, days	20.7 (27.49); 57	1.6 (4.28); 59	1.9 (5.46); 58

Table 1 shows annualized data (12 month combined 16 weeks, 8 and 12 month data; 24 month combined 18 and 24 month data).

n **–** number of patients with data recorded. **°**Within 12 months prior to start of omalizumab treatment. *Total number of asthma-related healthcare uses was calculated if data for asthma-related hospitalizations, emergency room visits and unscheduled doctor visits were available. ^#^Excluded those patients for whom this category was not applicable. Conclusion: Results from the eXpeRience registry showed that omalizumab reduced healthcare utilization and the number of days missed from school or work by asthma patients in a real-world setting. Thus, omalizumab treatment was associated with a positive and substantial impact on the direct and indirect costs linked with uncontrolled SAA.

